# Construction of a fatty acid metabolism-related gene signature for predicting prognosis and immune response in breast cancer

**DOI:** 10.3389/fgene.2023.1002157

**Published:** 2023-03-01

**Authors:** Li Qian, Yi-Fei Liu, Shu-Min Lu, Juan-Juan Yang, Hua-Jie Miao, Xin He, Hua Huang, Jian-Guo Zhang

**Affiliations:** ^1^ Department of Pathology, Affiliated Hospital of Nantong University, Nantong, China; ^2^ Department of Oncology, Shanghai Jiaotong University School of Medicine Xinhua Hospital, Shanghai, China

**Keywords:** breast cancer, gene expression, fatty acid metabolism, immune cells, prognosis

## Abstract

**Background:** Breast cancer has the highest incidence among malignant tumors in women, and its prevalence ranks first in global cancer morbidity.

**Aim:** This study aimed to explore the feasibility of a prognostic model for patients with breast cancer based on the differential expression of genes related to fatty acid metabolism.

**Methods:** The mRNA expression matrix of breast cancer and paracancer tissues was downloaded from The Cancer Genome Atlas database. The differentially expressed genes related to fatty acid metabolism were screened in R language. The TRRUST database was used to predict transcriptional regulators related to hub genes and construct an mRNA–transcription factor interaction network. A consensus clustering approach was used to identify different fatty acid regulatory patterns. In combination with patient survival data, Lasso and multivariate Cox proportional risk regression models were used to establish polygenic prognostic models based on fatty acid metabolism. The median risk score was used to categorize patients into high- and low-risk groups. Kaplan–Meier survival curves were used to analyze the survival differences between both groups. The Cox regression analysis included risk score and clinicopathological factors to determine whether risk score was an independent risk factor. Models based on genes associated with fatty acid metabolism were evaluated using receiver operating characteristic curves. A comparison was made between risk score levels and the fatty acid metabolism-associated genes in different subtypes of breast cancer. The differential gene sets of the Kyoto Encyclopedia of Genes and Genomes for screening high- and low-risk populations were compared using a gene set enrichment analysis. Furthermore, we utilized CIBERSORT to examine the abundance of immune cells in breast cancer in different clustering models.

**Results:** High expression levels of ALDH1A1 and UBE2L6 prevented breast cancer, whereas high RDH16 expression levels increased its risk. Our comprehensive assessment of the association between prognostic risk scoring models and tumor microenvironment characteristics showed significant differences in the abundance of various immune cells between high- and low-risk breast cancer patients.

**Conclusions:** By assessing fatty acid metabolism patterns, we gained a better understanding of the infiltration characteristics of the tumor microenvironment. Our findings are valuable for prognosis prediction and treatment of patients with breast cancer based on their clinicopathological characteristics.

## 1 Introduction

Breast cancer, a serious health threat to women worldwide, is the most common malignant tumor among women (Siegel et al., 2021). As of 2020, breast cancer accounts for 11.7% of all cancer cases worldwide, surpassing lung cancer as the leading cause of cancer incidence among women (Sung et al., 2021). A previous study showed that 416,317 cases of breast cancer were diagnosed in China in 2020, accounting for approximately 19.9% of the total number of female cancer patients (Fan et al., 2014). Breast cancer is a heterogeneous disease with widely varying molecular subtypes and clinical features. This feature not only poses a dilemma for the biological study of breast cancer but also a major challenge for its diagnosis and treatment (Dawson et al., 2013).

Fatty acid (FA) metabolism is one of the fundamental life activities in organisms and consists mainly of FA anabolism, FA β-oxidation, and lipid catabolism, forming an FA cycle (Kagawa et al., 2019). Part of the fatty acids in the body come from exogenous pathways, while part of them come from endogenous pathways involving acetyl CoA, which is produced through carbohydrate oxidation and proteolytic metabolism. The synthesized fatty acids can be further converted into triglycerides (TG) and lipid droplets (LD) for energy storage in addition to being used for membrane lipid synthesis. It is believed that TG undergoes β-oxidation processes in order to prevent the accumulation of intracellular lipids and lipotoxicity and to provide ATP and NADPH under metabolic stress conditions (Monaco, 2017; Petan et al., 2018; Olzmann and Carvalho, 2019). A variety of cancers promote rapid tumor growth by upregulating fat intake, storage, and adipogenesis (Abramson, 2011; Schulze and Harris, 2012; Röhrig and Schulze, 2016; Viswanathan et al., 2017). As a means of surviving stressful microenvironment conditions, multiple cancer cells rely on acetyl CoA to proliferate, metastasize, and resist stress. In addition to providing energy supply for the tricarboxylic acid cycle, acetyl CoA upregulates fatty acid synthesis to create favorable conditions for cell survival, suggesting that lipid metabolism plays a crucial role in cancer cell survival (Corbet and Feron, 2017). *De novo* FA synthesis plays a crucial role in diseases such as cancer, neurogenesis, and metabolic syndrome (Svensson et al., 2016). For example, deletion of chromosome 8p in breast cancer activates FA synthesis, suggesting that FA synthesis is essential for the development and progression the disease (Cai et al., 2016).

Despite the importance of FAs in tumorigenesis and tumor progression, lipid metabolic remodeling in breast cancer has not received the same attention as in other cancers, and reports of abnormal FA metabolism based on bioinformatics are extremely limited. Using the publicly available The Cancer Genome Atlas (TCGA) database, we downloaded mRNA expression matrices from breast tumor and paracancer tissues. Bioinformatics methods were used to screen differentially expressed FA metabolism-related genes and intersections to generate common FA metabolic pathway-related differentially expressed genes (DEGs).

Oncotype DX, MammaPrint, and Genomic Grade Index are first-generation prognostic markers for breast cancer; Prosigna, Endoppredict, and Breast Cancer Index are second-generation prognostic markers (Győrffy et al., 2015; Saini et al., 2019). Oncotype DX is one of the most widely used genetic markers for predicting the prognosis of breast cancer. Through real-time polymerase chain reaction, Oncotype DX can determine whether chemotherapy is needed in patients at low risk of relapse and predict the probability of disease recurrence (Vieira and Schmitt, 2018). The tumor immune microenvironment has been extensively studied to identify novel prognostic and predictive biomarkers (Soysal et al., 2015; Tray et al., 2018; Baxevanis et al., 2019).

Research has identified the tumor microenvironment (TME) as a major determinant of cancer growth (Li et al., 2007). The TME comprises several components, including inflammatory cells, stromal tissue (immune cells, fibroblasts, cytokines), and extracellular matrix (Witz and Levy-Nissenbaum, 2006). As cancer progresses from the initial transformation stage to invasion and metastasis, changes in the TME become increasingly important (Oudin and Weaver, 2016). It is not only one of the major factors triggering tumor progression but also one of the major challenges for effective immunotherapy, as non-malignant cells can encourage tumor cell proliferation, invasion, and metastasis within the TME (Jiang et al., 2019; Liu et al., 2019). An intricate series of pathways leads to tumorigenesis as immune cells coexist and interact. Approximately 70%–80% of the immune cells in the breast cancer microenvironment are T lymphocytes, with the remaining consisting of B lymphocytes, macrophages, natural killer (NK) cells, and antigen-presenting cells (Schmid et al., 2018; Adams et al., 2020; Schmid et al., 2020). In the regulation of immune response, fatty acid metabolism is a key metabolic pathway. Aside from providing energy to immune cells, it also acts as a precursor and substrate for the synthesis of cellular components and signaling molecules. In comparison to glycolysis and amino acid metabolism, fatty acid metabolism has been relatively little studied due to the complexity of fatty acid species, differences in the metabolism of exogenous and endogenous fatty acids, and insufficient detection methods (Li and Zhang, 2016).

In this study, using TCGA database, a polygenic prognosis model based on FA metabolism was developed for breast cancer. A comprehensive evaluation was also performed on the relationship between the prognostic risk scoring model and TME characteristics. Our understanding of the infiltration characteristics of the TME can be improved by evaluating FA metabolic patterns. The results suggest that personalized clinical diagnosis and treatment plan can be presented for high-risk groups based on the clinicopathological characteristics of patients.

## 2 Materials and methods

### 2.1 Data sources

Data from RNA-seq techniques (HTSeq-FPKM and HTSeq-counts) along with clinical information from 1,109 breast cancers and 113 normal samples were obtained from TCGA Genomic Data Commons portal (https://portal.gdc.cancer.gov/). Prognostic data were obtained from Liu et al. (2018). A total of 158 hallmark genes of FA metabolism were derived from the Molecular Signatures database (Liberzon et al., 2015).

### 2.2 Analysis of genes with differential expression

For the analysis of DEGs, we used the DESeq2 package (Love et al., 2014) with |logFC| >1 and adjusted *p*-value < 0.05 as the threshold. The results of the variance analysis were visualized on volcano plots and heatmaps using the ComplexHeatmap package (Gu et al., 2016).

### 2.3 Construction of molecule–molecule interaction networks

The STRING database combines experimental data, data generated from text mining PubMed abstracts, data synthesized from other databases, and results predicted using bioinformatics (Szklarczyk et al., 2021). Using the CytoHubba plug-in, the maximum clique centrality (MTT) values were calculated for DEGs based on the STRING database (Chin et al., 2014). Genes with the highest maximum clique centrality values were selected as hub genes. The GOSemSim package (Yu et al., 2010) was used to perform a Friends analysis on FA-related DEGs, ranking them in order of their interactions with genes from other pathways. We defined a set of parameters to predict miRNAs of hub-genes, including miRNAs with target sites in the 3′UTR region, a score above 0.95, and the Mirtarbase platform. TRRUST is a database containing 8,444 and 6,552 transcription factor (TF)–target regulatory relationships for 800 human and 828 mouse TFs, respectively (Han et al., 2018). We predicted the transcriptional regulators related to hub genes using the TRRUST data to construct an mRNA–TF interaction network.

### 2.4 Enrichment analysis

Functional enrichment studies typically use a gene ontology (GO) analysis approach, which includes determination of biological processes, molecular functions, and cellular components (Ashburner et al., 2000). Genomes, biological pathways, diseases, and drugs were all obtained from the Kyoto Encyclopedia of Genes and Genomes (KEGG) (Kanehisa et al., 2022). The FA-related DEGs were analyzed using ClusterProfiler (Yu et al., 2012), which includes GO annotation and KEGG pathway enrichment analyses. A *p*-value < 0.05 was considered statistically significant.

### 2.5 Consensus clustering analysis

Resampling-based consensus clustering identifies each member and determines the number of subgroups in the cluster. Using the ConsensusClusterPlus package (Wilkerson and Hayes, 2010), a consensus clustering approach was used to identify different FA regulatory patterns based on related DEGs. The number of iterations in clustering analysis was set to 100.

### 2.6 Prognostic model construction

We used Lasso and Cox regressions based on the glmnet (Friedman et al., 2010) packages to estimate the correlation between FA-related DEGs and survival status in breast cancer. To calculate the prognostic value of the Lasso regression model, we plotted time-dependent receiver operating characteristic curves to calculate the area under the curve, along with 1,000 cross-validations.

### 2.7 Gene set enrichment analysis (GSEA)

GSEA is a computational method that analyzes whether genes are statistically different between two biological states (Subramanian et al., 2005). We used GSEA to investigate differences in biological processes between samples with high- and low-risk prognoses. An expression dataset is commonly used to estimate pathway and biological process changes. For GSEA analysis, we downloaded “c2. cp.v7.2. symbols.gmt [Curated]” from the Molecular Signatures database. A *p*-value < 0.05 was considered significant.

### 2.8 Immune infiltration analysis

By employing CIBERSORT (an R package) (Newman et al., 2015), we estimated the proportion of 22 tumor immune infiltration cells in different clustered clustering models using immunoassays: naïve B cells, memory B cells, plasma cells, CD8^+^ T cells, naïve CD4^+^ T cells, resting CD4^+^ memory T cells, activated CD4^+^ memory T cells, T follicular helper cells, regulatory T cells, γδ T cells, resting and activated NK cells, monocytes, macrophages (M0, M1, and M2), resting and activated dendritic cells, resting and activated mast cells, eosinophils, and neutrophils.

### 2.9 Statistical analyses

**FIGURE 1:**
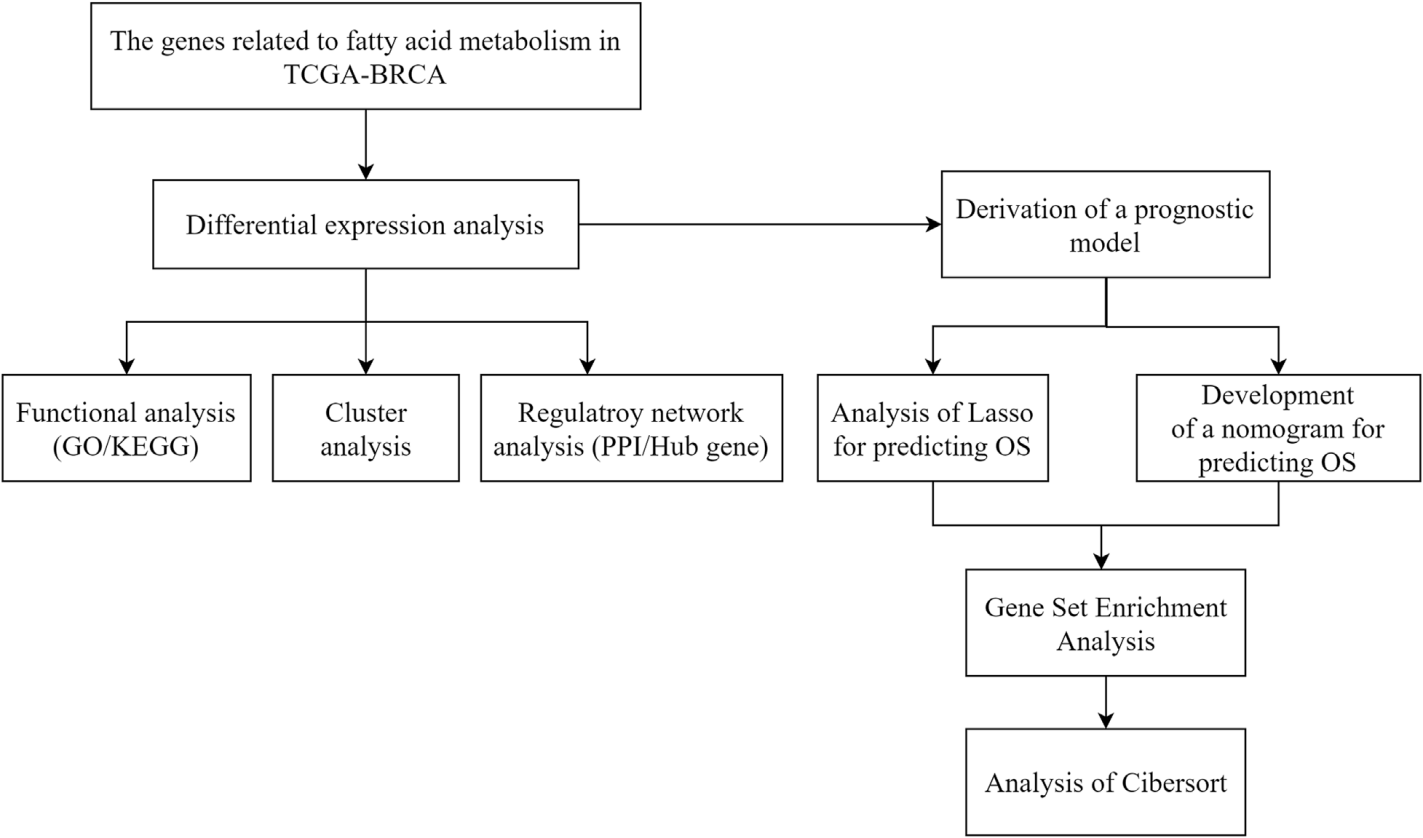
Technology roadmap for fatty acid metabolism-related gene prognostic models in breast cancer.

Statistical significance was determined for normally distributed variables using a *t*-test and for non-normally distributed variables using a Mann–Whitney *U* test (Silverman rank sum test). Statistical significance was assessed between the two categorical groups using Fisher’s exact test or chi-square test. *p* < 0.05 was considered statistically significant. The flow diagram in Figure 1 illustrates the overall process.

## 3 Results

### 3.1 Analysis of DEGs

**FIGURE 2:**
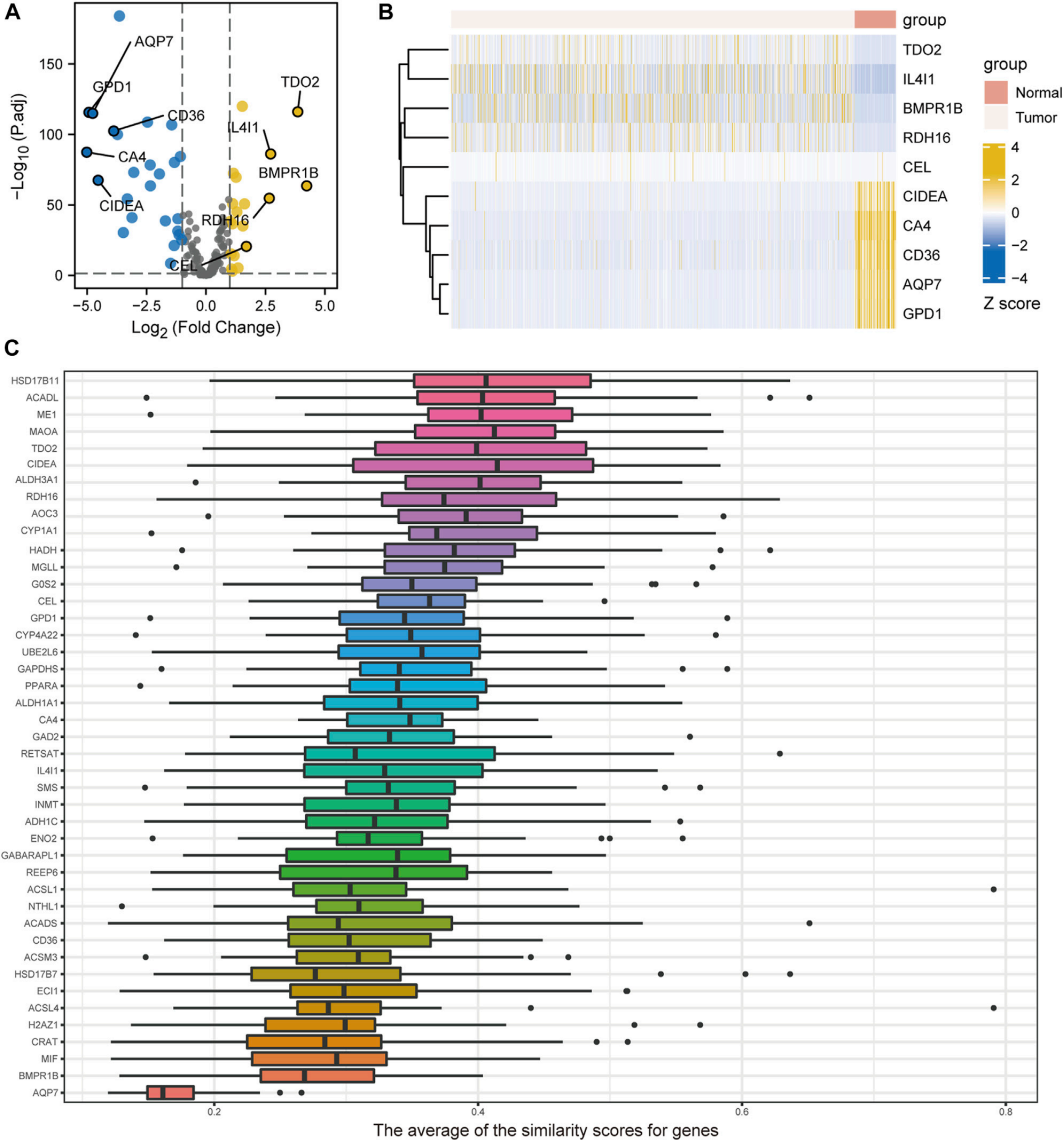
Differentially expressed genes (DEGs) related to fatty acid metabolism. **(A)** Volcano plot of TCGA-BRCA fatty acid metabolism-related genes in breast cancer and control samples. **(B)** Heatmaps illustrating differential expression of fatty acid metabolism-related genes from breast cancer and control samples from the TCGA-BRCA study. **(C)** Friends-based analysis demonstrating fatty acid metabolism-related DEGs.

The volcano plots of DEGs related to FA metabolism in breast cancer tissues and normal tissues are shown in Figure 2A. A total of 44 DEGs met the |log2(FC)| >1 threshold with a *p* < 0.05. Of these DEGs, 19 were highly expressed in tumors, including *BMPR1B*, *TDO2*, *IL4I1*, *RDH16*, and *CEL*. In normal tissues, *CD36*, *CIDEA*, *AQP7*, *GPD1*, and *CA4*, as well as 25 other genes, were highly expressed. The gene expression heatmap of the top five high- and low-expression genes in breast cancer is shown in Figure 2B. *HSD17B7*, *ACADL*, *ME1*, *MAOA*, and *TDO2* are the five genes related to FA metabolism with the largest differential expression, based on Friends analysis (Figure 2C).

### 3.2 Related regulatory network construction

**FIGURE 3:**
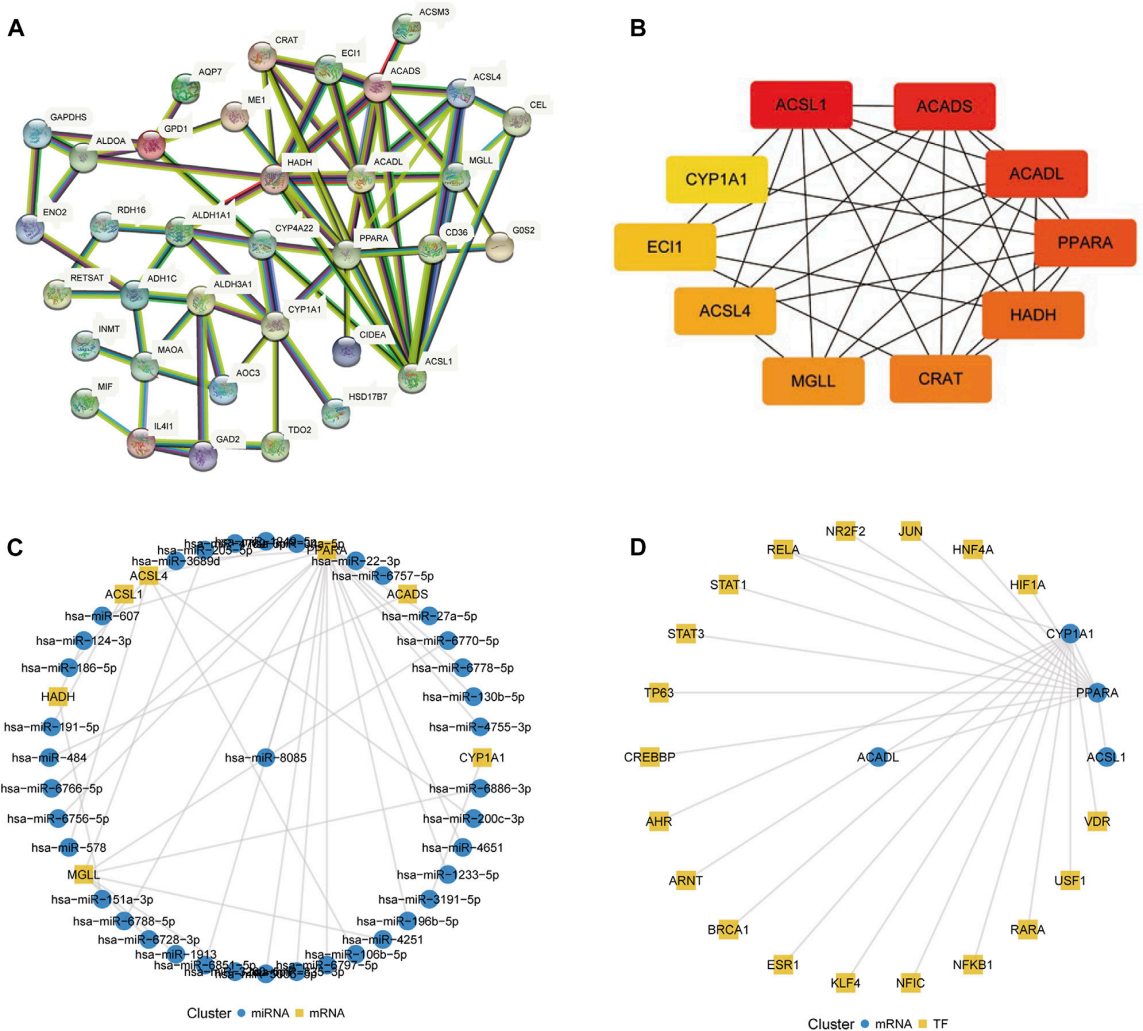
Construction of regulatory networks. **(A)** Protein–protein interaction network of fatty acid metabolism-related differentially expressed genes constructed using the STRING database. **(B)** Ten hub genes selected using the CytoHubba plugin. The color represents the gene MCC score, which ranges from 8 to 369, where the redder the color the higher the MCC score and the more orange the color the lower the MCC score. **(C)** mRNA–miRNA network constructed using the miRWalk database. **(D)** mRNA–transcription factor network constructed using the TRRUST database.

The protein–protein interaction (PPI) network of FA metabolism-related DEGs based on the STRING database is shown in Figure 3A. Based on maximum clique centrality values, 10 hub genes were identified: *ACSL1*, *ACADS*, *ACADL*, *PPARA*, *HADH*, *CRAT*, *MGLL*, *ACSL4*, *ECI1*, and *CYP1A1* (Figure 3B)*.* For these genes, miRNAs and transcriptional regulators were predicted to construct the mRNA–miRNA (Figure 3C) and mRNA–TF interaction networks (Figure 3D), respectively. Among them, transcription factors BRCA1, ESR1, VDR, STAT1, RARA, USF1, RELA were significantly more highly expressed in breast cancer than in normal tissues, and CREBBP, TP63, AHR, NFKB1, KLF4, NFIC, ARNT, JUN, PPARA were significantly less expressed in breast cancer than in normal tissues (Supplementary Figure S1).

### 3.3 Functional enrichment analysis

**FIGURE 4:**
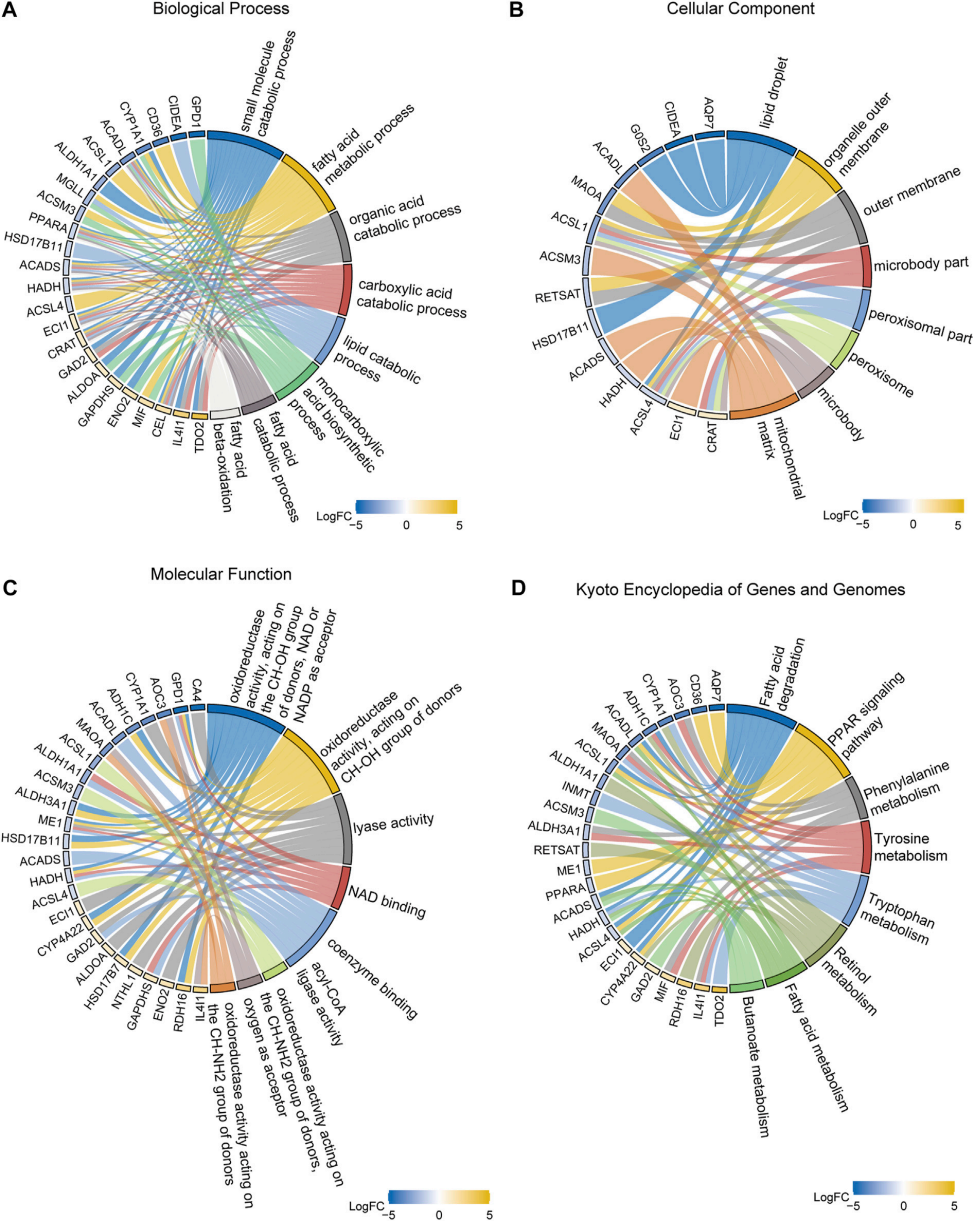
Gene ontology (GO) and Kyoto Encyclopedia of Genes and Genomes (KEGG) enrichment analyses. **(A)** GO biological processes. **(B)** GO cellular components. **(C)** GO molecular functions. **(D)** KEGG pathways.

In order to identify DEGs related to FA metabolism in breast cancer, we conducted GO and KEGG enrichment analyses. GO analysis revealed that DEGs were mainly involved in small molecule catabolism (Supplementary Table S1, Figure 4A). Enriched cellular components included lipid droplets, organelle outer membranes, outer membranes, microbody parts, peroxisomal parts, peroxisomes, microbodies, and mitochondrial matrix (Figure 4B). As for the molecular functions, NAD and NADP were the most prevalent acceptors for oxidoreductases, lyases, NAD binding, coenzyme binding, acyl-CoA ligases, and oxidoreductases acting on CH-NH_2_ groups of donors (Figure 4C). Based on KEGG functional analysis, DEGs were mostly affected by pathways such as FA metabolism and degradation; PPAR signaling pathway; phenylalanine, tyrosine, and tryptophan metabolism; retinol metabolism; and butanoate metabolism (Figure 4D, Supplementery Figure S2, Supplementary Table S2).

### 3.4 Analysis of FA metabolism-related DEGs for discovery of molecular isoforms

**FIGURE 5:**
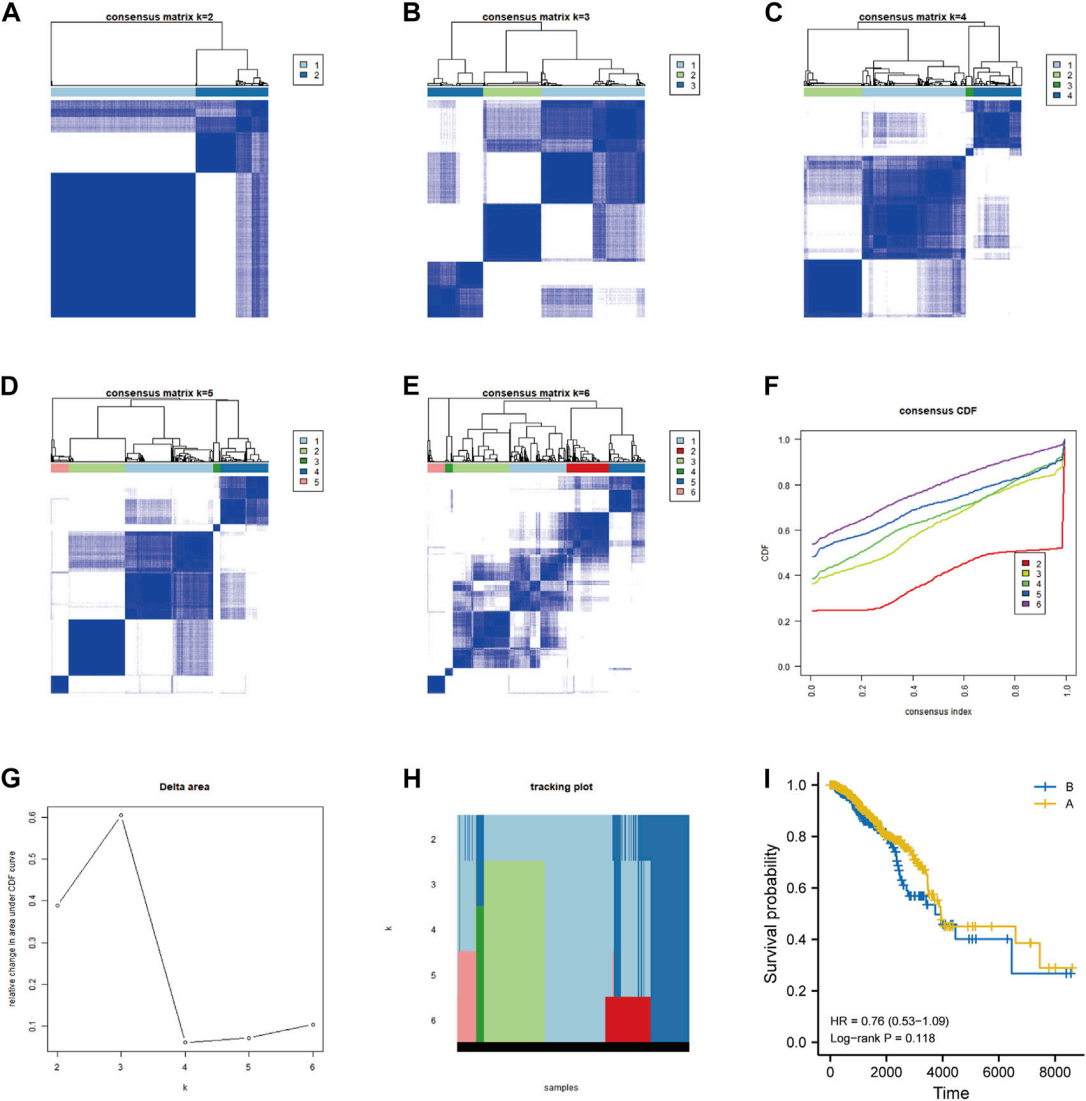
Determination of the molecular subtypes of breast cancer based on differentially expressed fatty acid metabolism-related genes. **(A–H)** Unsupervised consensus clustering analysis of breast cancer samples based on the differential expression of fatty acid metabolism-related genes. **(I)** Survival prognosis analysis of samples clustered into 2-class subtypes.

**FIGURE 6:**
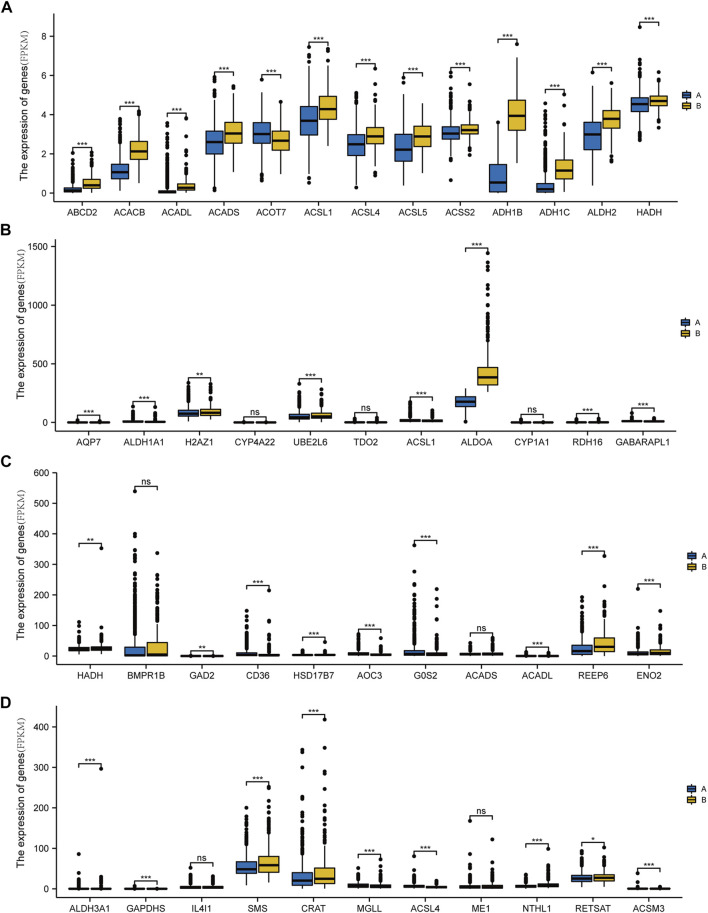
Differential expression of genes involved in fatty acid metabolism in different types of breast cancer. **(A)** Expression of *ADH1C*, *NMT*, *MIF*, *HSD17B11*, *MAOA*, *PPARA*, *CIDEA*, *CEL*, *ECI1*, *GPD1*, and *CA4* in breast cancer subtype A and B groups. **(B)** Expression levels of *AQP7*, *ALDH1A1*, *H2AZ1*, *CYP4A22*, *UBE2L6*, *TDO2*, *ACSL1*, *ALDOA*, *CYP1A1*, *RDH16*, and *GABARAPL1* in subtype A and B groups of breast cancer. **(C)** Export of *HADH*, *BMPR1B*, *GAD2*, *CD36*, *HSD17B7*, *AOC3*, *G0S2*, *ACADS*, *ACADL*, *REEP6*, and *ENO2* in subtype A and B groups of breast cancer. **(D)** *ALDH3A1*, *GAPDHS*, *IL4I1*, *SMS*, *CRAT*, *MGLL*, *ACSL4*, *ME1*, *NTHL1*, *RETSAT*, and *ACSM3* expression in the breast cancer subtype A and B groups. (ns, *p* ≥ 0.05; *, *p* < 0.05; **, *p* < 0.01; ***, *p* < 0.001).

On the basis of the differential expression of genes related to FA metabolism in breast cancer samples, we conducted an unsupervised consensus clustering analysis to examine regulatory mechanisms and prognosis. The results are shown in Figures 5A–H. The survival prognosis was further analyzed using DEGs related to FA metabolism in breast cancer clustered in two categories; group B had a lower survival prognosis than that of group A, which had a 10-year survival rate (Figure 5I, Supplementary Table S5). Figure 6 illustrates the DEGs involved in FA metabolism among breast cancer subtypes in groups A and B.

### 3.5 Prognostic model construction

**FIGURE 7:**
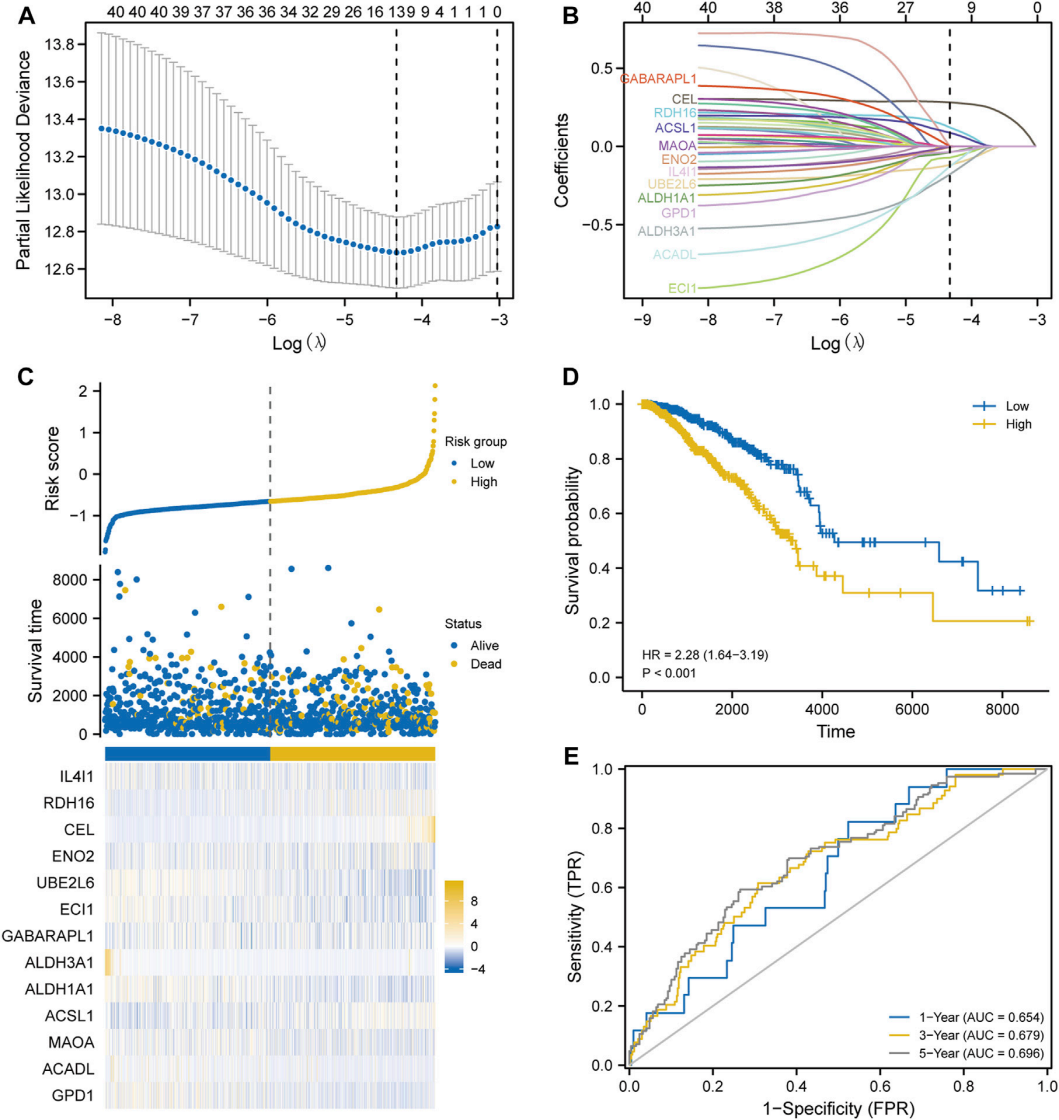
Modeling prognosis using Lasso analysis. **(A)** Determination of the Lasso coefficient. **(B)** Lasso variable trajectory plot. **(C)** Risk factor plot. **(D)** Survival analysis. **(E)** Receiver operating characteristic curve.Z.

**FIGURE 8:**
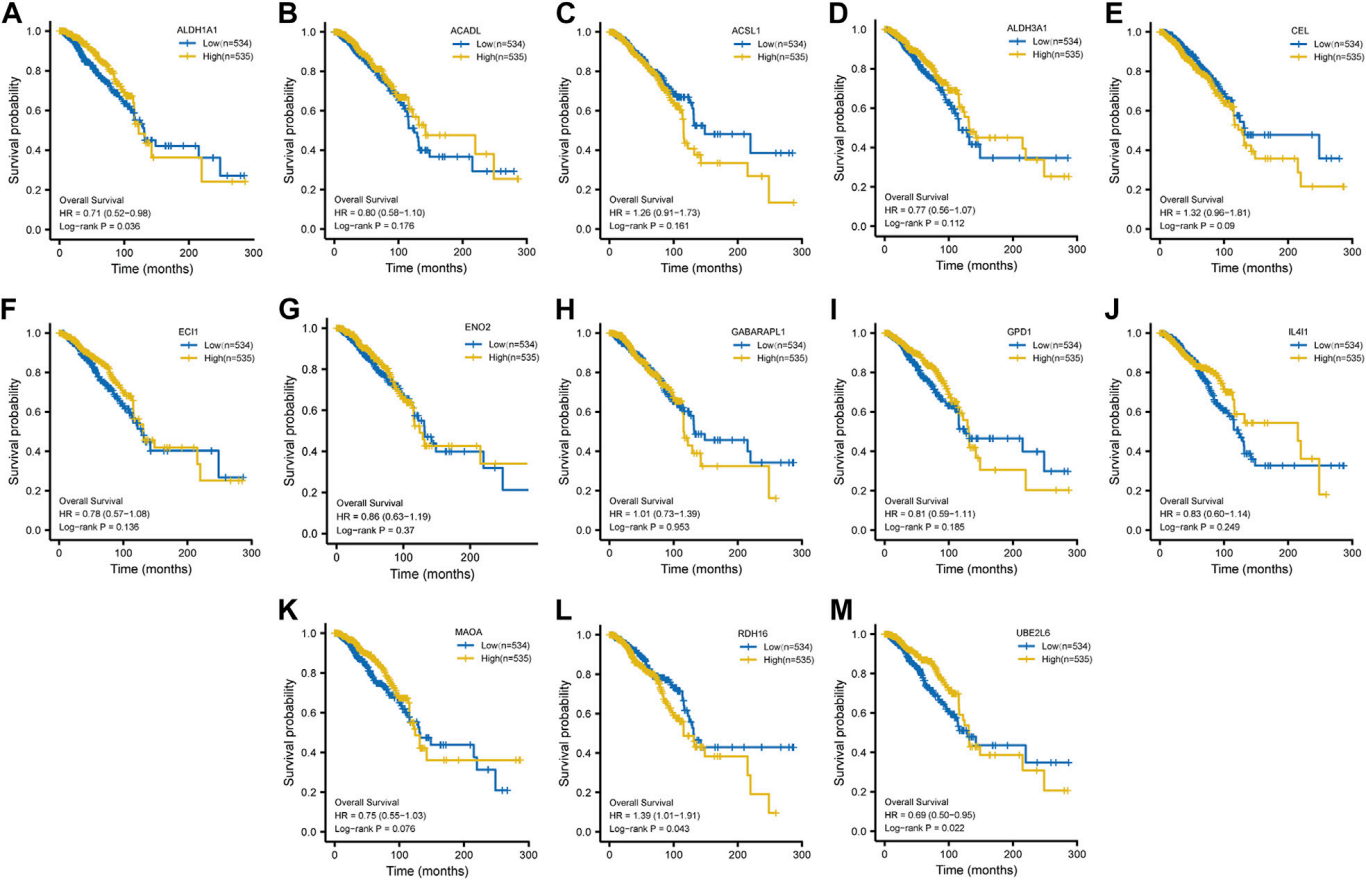
Kaplan–Meier survival analysis. Survival curves of *ALDH1A1* **(A)**, *ACSL1* **(B)**, *ALDH1A1* **(C)**, *ALDH3A1* **(D)**, *CEL* **(E)**, *ECI1* **(F)**, *ENO2* **(G)**, *GABARAPL1* **(H)**, *GPD1* **(I)**, *IL4I1* **(J)**, *MAOA* **(K)**, *RDH16* **(L)**, and *UBE2L6* **(M)**.

**FIGURE 9:**
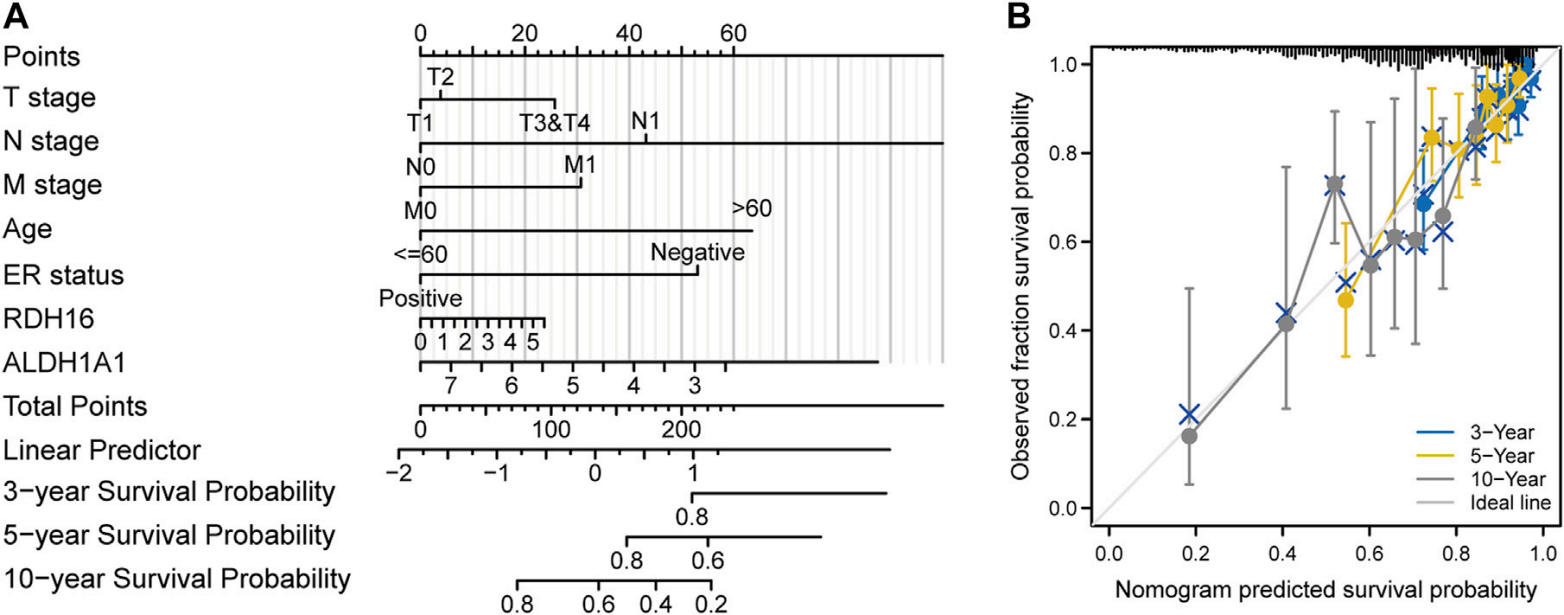
Construction of a prognostic model. **(A)** Column line graph. **(B)** Calibration curve.

After Lasso analysis of 44 FA metabolism-related DEGs, the prognostic risk score model was constructed using 13 genes: *IL4I1*, *RDH16*, *CEL*, *ENO2*, *UBE2L6*, *ECI1*, *GABARAPL1*, *ALDH3A1*, *ALDH1A1*, *ACSL1*, *MAOA*, *ACADL*, and *GPD1* (Figures 7A–C). Furthermore, the area under the time-dependent receiver operating characteristic curve indicated that the accuracy of this prognostic model increased with increased survival time (Figures 7D, E). After further analysis of the clinical data from the included studies (Supplementary Table S3), Kaplan–Meier survival analysis revealed that high expression levels of both *ALDH1A1* and *UBE2L6* were protective against breast cancer (HR 0.71 and 0.69, respectively, *p* < 0.05), whereas high expression levels of *RDH16* were associated with a high risk of developing the disease (HR = 1.39, *p* < 0.05) (Figure 8). Univariate and multifactorial Cox results (Supplementary Table S4) showed that age (*p* < 0.001), estrogen receptor (ER) positive status (*p* = 0.042), and *RDH16* expression (*p* = 0.026) were independently associated with poor outcomes for TCGA-BRCA patients. By combining the results of Cox analysis and the clinical characteristics, we constructed a prognostic model and evaluated the risk probabilities (Figure 9). The calibration curves indicate that the model is predictive for 3-, 5-, and 10-year prognoses.

### 3.6 GSEA

**FIGURE 10:**
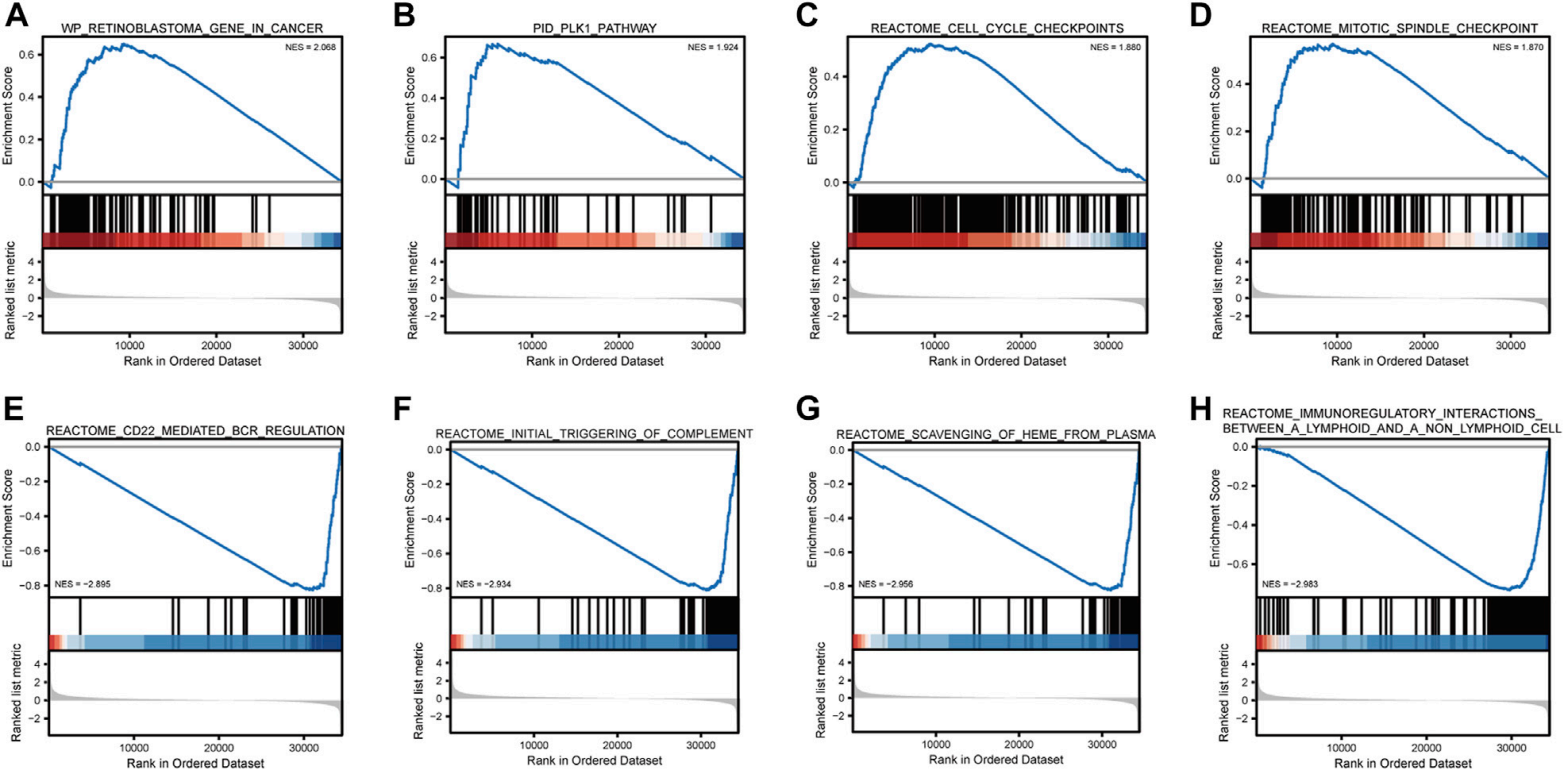
Gene set enrichment analysis. Breast cancer is mainly enriched in WP retinoblastoma gene in cancer **(A)**, PID PLK1 pathway **(B)**, reactome cell cycle checkpoints **(C)**, reactome mitotic spindle checkpoint Biocarta MCM pathway **(D)**, reactome CD22-mediated BCR regulation **(E)**, reactome initial triggering of complement **(F)**, reactome scavenging of heme from plasma **(G)**, and reactome immunoregulatory interactions between lymphoid and non-lymphoid cells **(H)**.

GSEA showed that retinoblastoma, PLK1 pathway, cell cycle checkpoints, and mitotic spindle checkpoint were significantly enriched in high-risk breast cancer (Table 1; Figure 10). The current analysis is a GSEA analysis based on all genes and corresponding logFCs from the differential analysis of high-risk vs. low-risk breast cancers, and when the NES is positive, the gene set is enriched in the high-risk group and *vice versa* in the low-risk group. CD22-mediated B-cell receptor (BCR) regulation, initial triggering of complement, scavenging of heme from plasma, and immunoregulatory interactions between lymphoid–nonlymphoid cell pathways were significantly enriched in low-risk breast cancer.

**TABLE 1:** Gene sets enriched in high-phenotype traits.

Description	setSize	enrichmentScore	NES	*P*-value
WP_RETINOBLASTOMA_GENE_IN_CANCER	86	0.6502	2.0679	0.0013
PID_PLK1_PATHWAY	46	0.6670	1.9241	0.0014
REACTOME_CELL_CYCLE_CHECKPOINTS	256	0.5227	1.8798	0.0012
REACTOME_MITOTIC_SPINDLE_CHECKPOINT	111	0.5689	1.8696	0.0013
REACTOME_CD22_MEDIATED_BCR_REGULATION	61	−0.8261	−2.8955	0.0034
REACTOME_INITIAL_TRIGGERING_OF_COMPLEMENT	79	−0.8116	−2.9339	0.0040
REACTOME_SCAVENGING_OF_HEME_FROM_PLASMA	69	−0.8247	−2.9559	0.0036
REACTOME_IMMUNOREGULATORY_INTERACTIONS_BETWEEN_A_LYMPHOID_AND_A_NON_LYMPHOID_CELL	185	−0.7328	−2.9830	0.0053

### 3.7 Immune infiltration analysis

**FIGURE 11:**
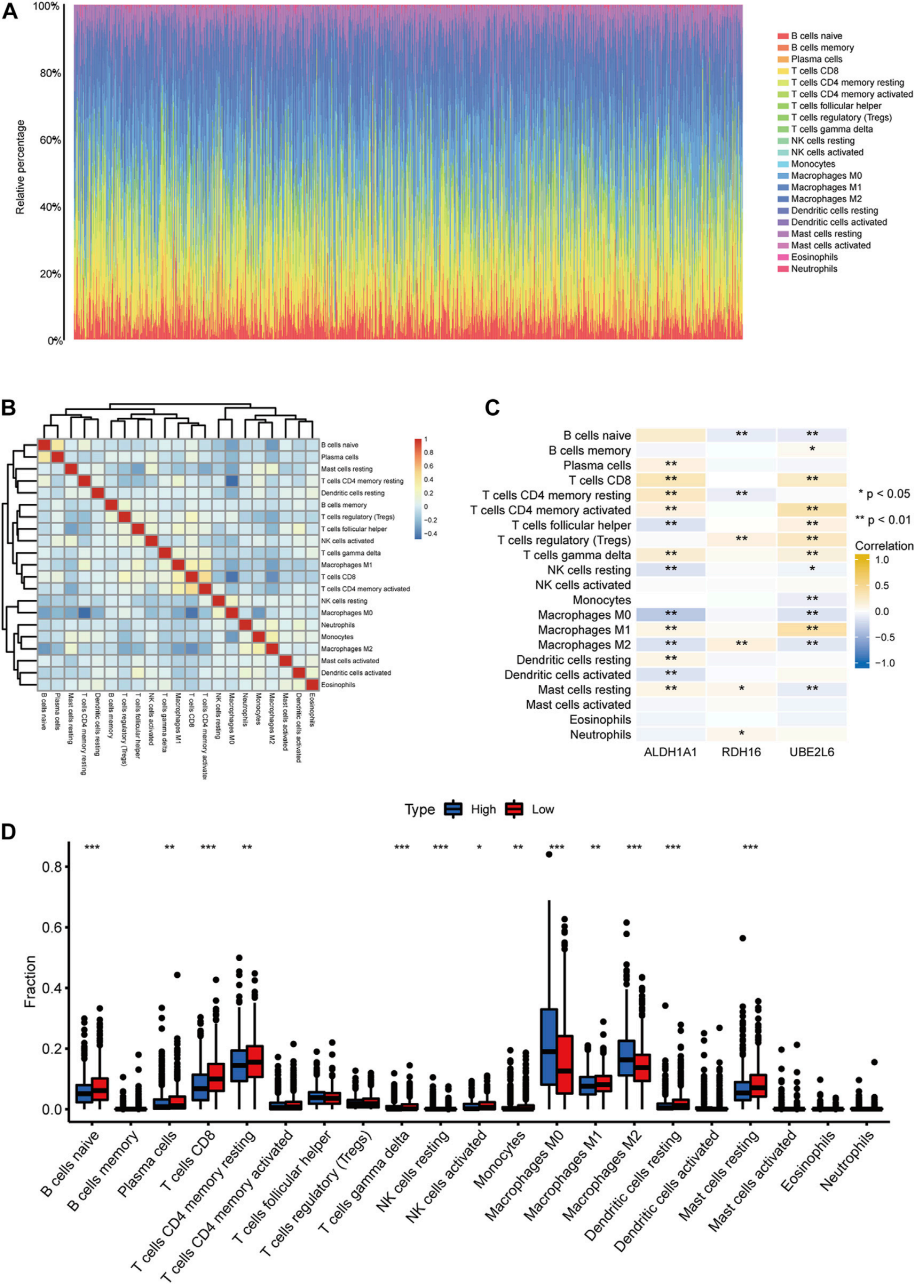
Immune infiltration analysis. **(A)** Abundance of immune cells in breast cancer samples. **(B)** Correlation heatmap of immune cells in breast cancer samples. **(C)** Correlation heatmap between differentially expressed fatty acid metabolism-related genes and immune cell infiltration levels. **(D)** Abundance of immune cells in high- and low-prognostic risk groups in breast cancer. (ns, *p* ≥ 0.05; *, *p* < 0.05; **, *p* < 0.01; ***, *p* < 0.001).

CIBERSORT was used to assess immune cell abundance in breast cancer samples (Figure 11A), correlations between immune cell abundance and prognosis- and FA-related DEGs in breast cancer (Figures 11B, C), and abundance of immune cells in prognostic high- and low-risk breast cancers (Figure 11D). *ALDH1A1* expression levels correlated significantly with plasma cells, CD8 T cells, resting and activated CD4 memory T cells, γδ T cells, M1 macrophages, resting dendritic cells, and resting mast cells. Infiltrates of T follicular helper cells, resting NK cells, M0 and M2 macrophages, and dendritic cells were significantly and negatively correlated with *ALDH1A1* expression levels.

## 4 Discussion

The incidence rate of breast cancer has exceeded that of lung cancer, ranking first worldwide, accounting for 11.7% of all new cancer cases (Sung et al., 2021). Globally, breast cancer is a serious threat to women’s health and lives (Chen et al., 2018; Siegel et al., 2021). It is a highly heterogeneous disease with unique biological characteristics and clinicopathological features among its molecular subtypes (Ng et al., 2017). Breast cancer has been classified into four main molecular subtypes based on microsequencing and gene expression profiling: luminal A, luminal B, HER2-overexpressing, and basal-like (Sorlie et al., 2003). This heterogeneity not only poses a challenge to the biological study of breast cancer but also to its diagnosis and treatment (Dawson et al., 2013). According to Monaco et al.’s study of the pathways of fatty acid metabolism in breast cancer, including the relationship between glucose and glutamine metabolism, fatty acid metabolism sustains the growth and survival of breast cancer cells. Compared with receptor-positive breast cancers (RPBC), triple-negative breast cancers (TNBC) appear more reliant on exogenous fatty acids uptake and storage. As well as being more heavily reliant on glucose (SLC2A1) and glutamine (SLC6A14) uptake, RPBC significantly upregulates *de novo* fatty acid synthesis, mobilization, and oxidation (Monaco, 2017). A significant difference in relative mRNA expression levels of FAM proteins was also observed using receptor status as well as PAM50 classification. RPBC primarily synthesized and oxidized from scratch, whereas quadruple negative breast cancer (QNBC) predominantly ingested and stored exogenous fatty acids (Monaco, 2017). There is a growing consensus that traditional clinical methods that assess breast cancer prognosis based on patient age, tumor size, histological features, and number of positive lymph nodes around the axilla are no longer valid. Cancer development is influenced by genetic variation. In recent years, gene technology-based therapies have shown a wide range of potential applications in cancer treatment. Gene therapy for cancer must be tailored to the disease-related genetic characteristics or genetic variants of each individual to be efficient, produce the best results in the shortest time, and reduce the side effects. The search for novel therapeutic targets, construction of genetic prediction models for the prognosis of patients, and improvement of prognosis through new site-targeted therapies have been hot topics in the field of breast cancer treatment research.

FA signaling and metabolism are among the most important pathways in tumor development, as lipids serve as an important source of energy during tumorigeneses (Kagawa et al., 2019). Recent research indicates that lipid metabolism is reprogrammed in several tumors, providing energy storage, intermediates, and even a major energy source for tumor proliferation, metastasis, and progression (Koundouros and Poulogiannis, 2020). Menendez and Lupu (2007) have suggested that tumor cells synthesize FAs autonomously to maintain their rapid proliferation, providing them with a survival advantage. An elevated lipid metabolic flux in cancer cells is associated with altered lipid metabolic pathways, including FA uptake, synthesis, storage, and release. During cancer cell growth, elevated lipid flux may supply phospholipid synthesis substrate. Many cancer cells produce more phosphatidylcholine (PC), and choline kinases necessary for PC synthesis are expressed and active in breast, prostate, lung, ovarian, and colon cancers. (Ramírez de Molina et al., 2002; Iorio et al., 2010). A further finding is that tumor cells grow when choline kinase is overexpressed (Glunde et al., 2011). Cell transformation and growth may be limited by PC synthesis, and increased FA flux provides PC synthesis substrate (Chen and Li, 2016). Therefore, the study of FA metabolism in tumor cells and the development of related enzyme inhibitors has become increasingly important.

Studies on FA metabolism in breast cancer are scarce, and the related literature is very limited. In this study, using TCGA data, *HSD17B7*, *ACADL*, *ME1*, *MAOA*, and *TDO2* were identified as the top five DEGs related to FA metabolism in breast cancer tissues. The enzyme 17β-hydroxysteroid dehydrogenase type 7 (HSD17B7) is a 32-kDa microsomal protein that catalyzes estradiol synthesis. Shehu Aurora et al. discovered that HSD17B7 is highly expressed in human ductal and breast cancer cell lines and that estradiol strongly upregulates HSD17B7 expression in MCF-7 cells at the mRNA and protein levels (Shehu et al., 2011). Long-chain acyl coenzyme A dehydrogenase (ACADL) plays a key role in the catalysis and β-oxidation of branched-chain FAs (Wanders et al., 1998), as well as in the β-oxidation of mitochondrial unsaturated FAs (Lea et al., 2000). Mitochondrial dysfunction caused by ACADL deficiency can lead to hepatic steatosis and insulin resistance (Zhang et al., 2007). ACADL promotes prostate cancer cell growth, as well as malignant transformation, when expressed in prostate cancer tissues, with a positive correlation between malignancy and metastasis (Xie et al., 2010; Xie et al., 2011). The role of ACADL in the progression and development of breast cancer has not been studied. Malic enzyme 1 (ME1) catalyzes the conversion of malate into pyruvate while generating NADPH from NADP (Merritt et al., 2009; Rzezniczak and Merritt, 2012; Goodman et al., 2018). Studies have shown that ME1 expression is higher in breast cancer tissues than in adjacent non-tumor tissues (Liao et al., 2018). In tumor cells, decreased *ME1* gene expression or inhibition of ME1 activity results in decreased cell proliferation, epithelial–mesenchymal transition, and migration *in vitro*, which in turn promote oxidative stress, apoptosis, and/or cellular senescence (Simmen et al., 2020). Monoamine oxidase A (MAOA) is a mitochondrial enzyme found mainly in catecholaminergic neurons (Shih et al., 1999). Research has shown that MAOA promotes tumor invasion, migration, and epithelial–mesenchymal transition (Wu et al., 2014). Satram-Maharaj et al. (2014) showed that MAOA expression was increased in breast cancer cell lines and selective MAOA inhibitors altered the growth, migration, and invasion of anchored non-dependent growth of breast cancer cells. Liu et al. (2020) showed that overexpression of tryptophan 2,3-dioxygenase (TDO2) was positively correlated with breast cancer malignancy and tumor grade; the expression of TDO2 was higher in estrogen-negative and triple-negative breast cancers than in other subtypes and was associated with poorer prognoses in patients with breast cancer. Furthermore, they found that TDO2 contributes to the regulation of the immune microenvironment and tryptophan metabolism in breast cancer and is associated with poor prognoses. According to their findings, TDO2 may be a promising immunotherapeutic target for breast cancer. Our current study confirms these findings.

The development of tumors is a complex pathophysiological process regulated by intricate molecular mechanisms. We clarified how key DEGs in breast cancer contribute to the development of the disease by building a protein–protein interaction network as well as mRNA–miRNA and mRNA–TF interaction networks. *ACSL1*, *ACDS*, *ACADL*, *PPARA*, *HADH*, *CRAT*, *MGLL*, *ACSL4*, *ECI1*, and *CYP1A1* were identified as hub genes.

KEGG pathway enrichment analysis was performed on co-distinctively expressed genes to understand the molecular mechanisms of oncogenic effects and related pathways. KEGG pathway maps represent the current knowledge on molecular interactions and relationships of various biological processes. Genes in the genome are linked to gene products (mainly proteins) in pathways through molecular interactions and reactions. Therefore, KEGG pathway analysis can be used to determine crosstalks between pathways and associated functions in the genome. GO functional enrichment analysis revealed that co-upregulated DEGs were highly enriched in catabolic processes involving small molecules, FAs, lipids, organic acids, and carboxylic acid acids. The results suggested that key genes for the development of breast cancer were directly involved in the biological processes associated with FAs. Li et al. (2022) demonstrated that elevated fatty acid oxidation (FAO) activates STAT3 *via* acetylation of acetyl coenzyme A (CoA). The acetylation of STAT3 stimulates the expression of long-chain acyl coenzyme A synthase 4 (ACSL4), which increases phospholipid synthesis. Increasing mitochondrial phospholipid content increases mitochondrial integrity, which in turn prevents chemotherapy-induced tumor cell apoptosis. A decrease in mitochondrial membrane phospholipids was observed with enhanced apoptosis of cancer cells in cultured tumor cells and xenograft tumors following inhibition of ASCL4 or targeted acetylation of STAT3. Additionally, Migita et al. (2017) demonstrated that overexpression of long-chain acyl coenzyme A synthase 3 (ACSL3), which activates cholesterol synthesis and steroidogenesis, was downregulated in triple-negative breast cancers but overexpressed in androgen-dependent cancers (such as prostate tumors). In contrast, KEGG pathway enrichment analysis identified FA degradation; PPAR signaling pathway; phenylalanine, tyrosine, and tryptophan metabolism; retinol metabolism; FA metabolism; and butanoate metabolism, among other pathways. Breast cancer originates from abnormal and rapid growth of mammary epithelial cells. The above results suggest that the identified DEGs may regulate FA metabolism in breast cancer cells by affecting the above pathways, thus regulating the development and progression of breast cancer.

Unsupervised consensus clustering was conducted on breast cancer samples based on the expression of DEGs associated with FA metabolism to investigate the regulatory mechanisms of these genes and the prognosis. Based on the clustering of FA metabolism-related DEGs into two classes, we further analyzed the survival prognosis of patients. With the aim of investigating the role of 44 DEGs associated with FA metabolism on breast cancer survival, Lasso, Kaplan–Meier, and univariate and multivariate Cox regression analyses were performed. We identified 13 survival-associated genes involved in differential FA metabolism in this study, namely *IL4I1*, *RDH16*, *CEL*, *ENO2*, *UBE2L6*, *ECI1*, *GABARAPL1*, *ACSL1*, *MAOA*, *ACADL,* and *GPD1*. In order to test the independent prognostic value of survival- and FA metabolism-related DEGs, we performed independent prognostic analysis by combining clinical factors (such as gender, age, grade, and pathological stage) with genes and found that high expression levels of *ALDH1A1* and *UBE2L6* were protective against breast cancer. TCGA-BRCA patients with high expression of *RDH16* had a worse prognosis due to its independent prognostic value. The calibration curve revealed a relatively good predictive value for prognoses at 3, 5, and 10 years based on the model. The correlation between the results yielded by the model and clinicopathological factors verified an accurate prediction of prognoses for patients with breast cancer. In addition to catalyzing C10–C18 fatty acid oxidation, ACADL is an important enzyme in FA β-mono-oxidation. According to Zhao et al., ACADL expression is downregulated in hepatocellular carcinomas; such low levels of ACADL expression are associated with poor clinical prognoses in hepatocellular carcinoma (Zhao et al., 2020). Li et al. (2015) showed that ACADL methylation levels differed significantly among breast cancer subtypes and were associated with tumor ER status. Our study corroborates these findings.

We analyzed the abundance of immune cells in breast cancer samples to investigate the correlation between the presence of prognosis-related genes that affect FA metabolism and immune cell infiltration levels in breast cancer and the number of immune cells in prognostic high- and low-risk breast cancer samples. Known as epithelial heavy anisotropic hyperplasia, breast cancer evolves into *in situ*, invasive, and metastatic carcinomas (Polyak, 2007). Metastasis occurs when breast cancer cells spread to distant sites due to the loss of the myoepithelial layer and basement membrane after *in situ* cancer has progressed to invasive ductal carcinoma. Various types of cells in the TME play an important role in tumor progression and, therefore, are potential new therapeutic targets for breast cancer (Criscitiello et al., 2014; Bahrami et al., 2018). Low-risk breast cancers had significantly higher abundance of naïve B cells, plasma cells, CD8 T cells, and resting CD4 memory T cells and lower abundance of M0, M1, and M2 macrophages than high-risk breast cancers. CD4^+^ T cells have a dynamic role and subpopulation distribution in breast carcinogenesis and progression. According to a retrospective study on breast cancer, CD4^+^ T cells were positively related to tumor stage, size, and metastasis and negatively related to survival (Huang et al., 2015). As one of the most important players in the tumor microenvironment, tumor-infiltrating lymphocytes consist primarily of CD4 helper cells, CD4 cells, CD25 helper cells, regulatory T cells of the FOXP3 phenotype (Treg), and effector cells such as natural killer cells and CD8^+^ T cells. As Tregs suppress self-reactive T cells under normal conditions, they exert an immunosuppressive effect within the tumor microenvironment, allowing tumor cells to evade the immune system (Allen and Louise Jones, 2011; Mahmoud et al., 2011). Recent research has revealed that Treg can produce large amounts of RANKL, which promotes breast cancer metastasis through RANK expression (Tan et al., 2011). Accordingly, the above research shows that breast cancer patients with a high number of Treg have a worse prognosis (Bohling and Allison, 2008; Ohara et al., 2009). In breast cancer, tumor-associated macrophages promote tumor growth and angiogenesis, remodel tissues, and suppress adaptive immunity (Mantovani et al., 2008; Allen and Louise Jones, 2011). Breast cancer patients with high tumor-associated macrophage levels tend to have a poor prognosis, suggesting that depletion or reprogramming of these macrophages may be a viable therapeutic strategy (Tsutsui et al., 2005; Zhang et al., 2013), consistent with the results of our study. It is therefore necessary to conduct further research to confirm the mechanism of action of these features in the immune microenvironment.

Our study provides new insights into the role of FA metabolism in breast cancer; however, it has few limitations. First, patients with cancer are considerably more numerous than control subjects in TCGA database. In addition, public databases lack specific details about patient medications and/or surgical treatments, which can affect the assessment of their prognosis. Third, this study is mainly based on bioinformatics analysis of public databases for validation, and the authors’ team is collecting clinical samples and will use them for validation in further studies. As a final note, this is a retrospective study; prospective studies are necessary to compensate for its limitations.

In summary, breast cancer is a highly heterogeneous disease with multiple subtypes and different prognostic and therapeutic responses; these characteristics pose a great challenge to its treatment. In this study, we identified five key FA metabolism-related DEGs in breast cancer. Additionally, we successfully constructed an accurate prognostic risk score model using 13 DEGs related to FA metabolism for patients with breast cancer. Using FA metabolism-related prognostic genes as biomarkers in patients with breast cancer offers enhanced opportunities for accurate prognostics and provides a better understanding of the involved molecular mechanisms.

## Data Availability

The datasets presented in this study can be found in online repositories. The names of the repository/repositories and accession number(s) can be found in the article/Supplementary Material.

## Abbreviations

ACADL, long-chain acyl coenzyme A dehydrogenase; DEG, differentially expressed gene; FA, fatty acid; GO, gene ontology; GSEA, gene set enrichment analysis; HSD17B7, 17β-hydroxysteroid dehydrogenase type 7; KEGG, Kyoto Encyclopedia of Genes and Genomes; NK, natural killer; MAOA, monoamine oxidase A; ME1, malic enzyme 1; TCGA, The Cancer Genome Atlas; TDO2, tryptophan 2,3-dioxygenase; TF, transcription factor; TME, tumor microenvironment; BRCA, breast cancer.
