# Supplementary figures and images for: Construction of a fatty acid metabolism-related gene signature for predicting prognosis and immune response in breast cancer
Source: Front Genet. 2023 Mar 1;14:1002157. doi: 10.3389/fgene.2023.1002157 (PMC10014556; doi:10.3389/fgene.2023.1002157)

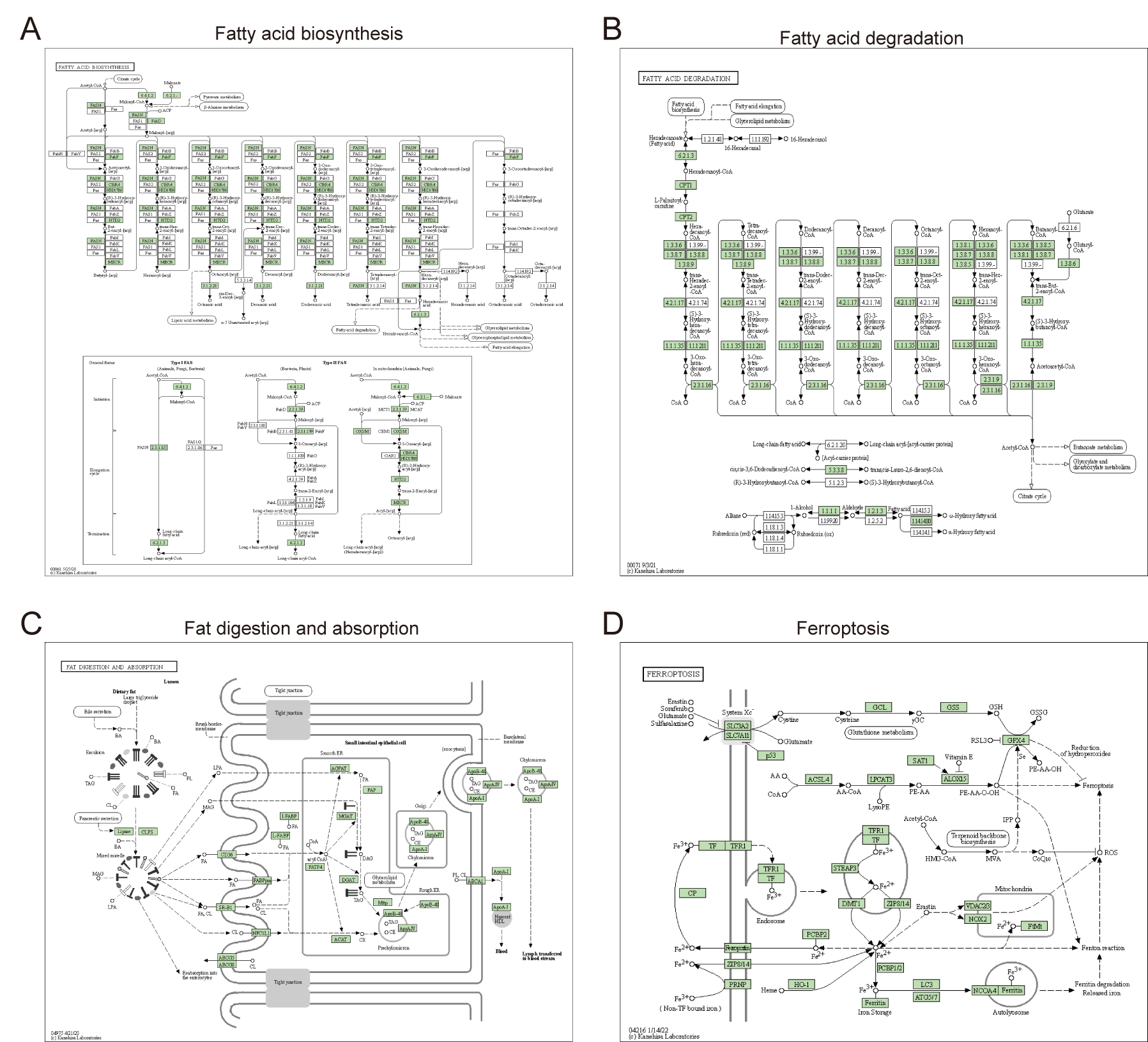

Supplement: Supplementary file 1 [file Image2.TIF]

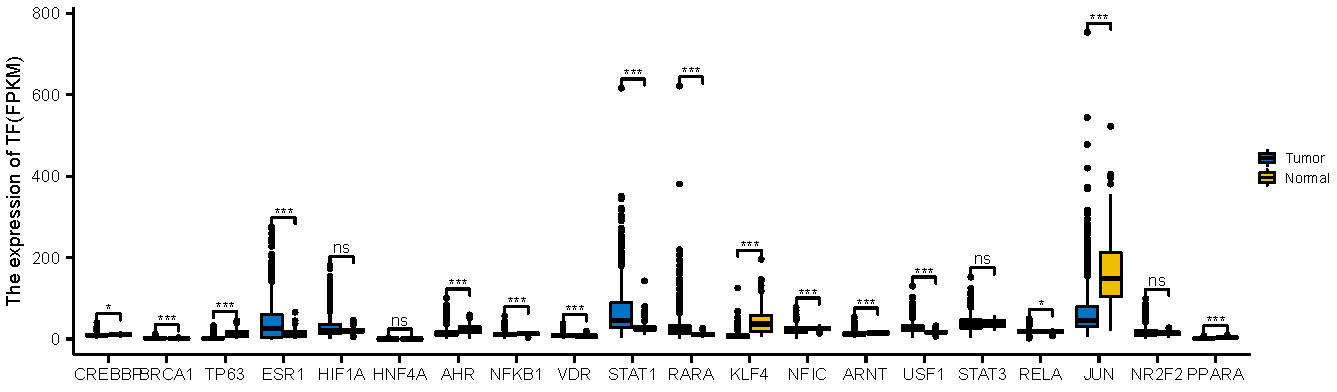

Supplement: Supplementary file 2 [file Image1.TIF]
